# Cardiac arrhythmias following COVID-19 vaccination: insights from the U.S. vaccine adverse event reporting system (VAERS), December 2020—January 2025, United States

**DOI:** 10.3389/fpubh.2026.1762082

**Published:** 2026-06-08

**Authors:** Yi Lu, Hao Jiang, Xiangwei Ding

**Affiliations:** Department of Cardiology, The Affiliated Taizhou People's Hospital of Nanjing Medical University, Taizhou School of Clinical Medicine, Nanjing Medical University, Taizhou, Jiangsu, China

**Keywords:** adenoviral vector vaccine, arrhythmia, COVID-19 vaccine, mRNA vaccine, VAERS

## Abstract

**Background:**

Although COVID-19 vaccines are highly effective in preventing infection and severe disease, their cardiovascular safety, particularly regarding arrhythmic risk, continues to raise concerns.

**Methods:**

A disproportionality analysis was conducted using the U.S. Vaccine Adverse Event Reporting System (VAERS) to evaluate arrhythmia-related AEs reported between December 2020 and January 2025. Adverse events (AEs) were identified using MedDRA Preferred Terms, and signal strength was assessed by Reporting Odds Ratio (ROR) and Proportional Reporting Ratio (PRR).

**Results:**

A total of 52,518 reports were identified, with a median onset interval of 1 day after vaccination. The most frequent AEs were tachycardia (40.5%) and atrial fibrillation (16.9%). Atrial flutter, extrasystoles, and atrioventricular block exhibited the strongest signals. Severe events primarily involved ventricular and conduction abnormalities, particularly among older adults and male individuals. Reports involving mRNA vaccines were predominantly characterized by tachyarrhythmia-related events, whereas findings for bradyarrhythmia- and conduction-related events after adenoviral vector vaccination should be interpreted cautiously.

**Conclusions:**

Cardiac arrhythmias following COVID-19 vaccination typically occurred within the early post-vaccination period and demonstrated clear platform- and population-specific differences. Although the overall risk is low, enhanced early monitoring and stratified pharmacovigilance are warranted to optimize vaccine safety.

## 1 Introduction

Since the emergence and global spread of severe acute respiratory syndrome coronavirus 2 (SARS-CoV-2) in 2019, coronavirus disease 2019 (COVID-19) has continued to exert profound impacts on public health and healthcare systems worldwide. In December 2020, the United States initiated a large-scale vaccination program, followed shortly by many other countries, aiming to reduce infection, severe illness, and mortality (1). Among various vaccine platforms, adenoviral vector and messenger RNA (mRNA) vaccines rapidly became predominant due to the short development timelines and design flexibility. Both platforms allow rapid adaptation to emerging variants through sequence modification, enabling swift responses and scalable production. Consequently, these two technologies have become central to global COVID-19 vaccine research and deployment (2). Therefore, vaccination remains the cornerstone of the public health response to mitigate disease burden and prevent severe outcomes.

As vaccination campaigns expanded, post-marketing surveillance and clinical observations have provided a clearer understanding of vaccine safety profiles. Most adverse events (AEs) reported after COVID-19 vaccination are mild, transient, and self-limiting, including injection-site pain or swelling, fatigue, headache, chills, myalgia, fever, arthralgia, nausea, and axillary lymphadenopathy (3–6). However, although rare, several serious AEs have drawn considerable clinical attention, such as anaphylaxis (7), vaccine-induced immune thrombotic thrombocytopenia (VITT/TTS) (8), immune thrombocytopenia (ITP) (9), Guillain-Barre syndrome (GBS) (10), Bell's palsy (11), and autoimmune hepatitis (12). Despite their low incidence, the potential severity and heightened public concern emphasize the need for ongoing pharmacovigilance and risk stratification.

Among various affected organ systems, cardiovascular safety has emerged as an issue of particular importance. Previous studies have suggested an association between COVID-19 vaccination and myocarditis [risk ratio (RR) 3.24, 95% confidence interval (CI) 1.55–12.44; risk difference 2.7 per 100,000 persons] (13). Although acute myocardial infarction following vaccination is relatively rare, its severity and potential fatality warrant vigilance (14). A nationwide retrospective cohort study in South Korea involving 3,350,855 vaccine recipients identified an increased risk of acute cardiac events within 21 days after the first dose (15). Similarly, a retrospective study conducted by Yan et al. demonstrated vaccine platform-dependent differences in signal strength and onset timing for severe cardiovascular AEs, including arrhythmias, hypertension, and heart failure (16). A systematic review reported that approximately 1.4% of cardiovascular complications following mRNA vaccination involved arrhythmias (17).

Cardiac arrhythmias refer to abnormalities in the initiation or conduction of cardiac electrical activity, leading to altered rate, rhythm, or conduction. They can be categorized by rate (tachyarrhythmias vs. bradyarrhythmias) or origin (supraventricular vs. ventricular). Common forms include atrial fibrillation, atrial flutter, supraventricular tachycardia, ventricular tachycardia, and ventricular fibrillation. These disorders are of major clinical significance, contributing substantially to morbidity and mortality. Without prompt recognition and management, arrhythmias can result in stroke, heart failure, or sudden cardiac death. Accordingly, early diagnosis and standardized management are critical (18).

Although the overall incidence of post-vaccination arrhythmias appears low (19), even a slight increase in risk could have public health implications given the vast global vaccinated population. Moreover, platform-specific differences in signal strength underscore the need for robust post-marketing surveillance. However, current studies on post-vaccination arrhythmias remain limited, mostly focusing on case reports or single-platform data, lacking systematic comparative analyses. Accordingly, we utilized data from the United States Vaccine Adverse Event Reporting System (VAERS) to systematically characterize arrhythmia-related AEs following different COVID-19 vaccines. This analysis aimed to generate data-driven hypotheses to guide future prospective studies and to strengthen evidence-based risk communication.

## 2 Methods

### 2.1 Study design and data source

VAERS is a national passive surveillance system jointly managed by the U.S. Centers for Disease Control and Prevention (CDC) and the Food and Drug Administration (FDA), primarily designed for the early identification of potential vaccine safety signals (20). We conducted a retrospective analysis based on the VAERS to evaluate reports of arrhythmia-related AEs following COVID-19 vaccination between December 2020 and January 2025 in the United States. As VAERS is a publicly available, de-identified database, this study qualifies as a secondary data analysis and did not require ethical approval.

### 2.2 Vaccine exposure

The following products were included as target exposures: “COVID19 (JANSSEN),” “COVID19 (MODERNA BIVALENT),” “COVID19 (MODERNA),” “COVID19 (PFIZER-BIONTECH BIVALENT),” and “COVID19 (PFIZER-BIONTECH).” If a report contained multiple vaccines, the presence of any target product was sufficient to classify it as exposed. In the disproportionality analysis, reports without any of the target vaccines served as the reference group.

### 2.3 Case definition and outcomes

Arrhythmia-related AEs were identified using Preferred Terms (PTs) from the Medical Dictionary for Regulatory Activities (MedDRA). The number, proportion, and signal strength of each PT were summarized at the overall level. PTs were further grouped into three composite outcomes: cardiac arrest-related events, tachyarrhythmia-related events, and bradyarrhythmia-related events.

### 2.4 Statistical analysis

#### 2.4.1 Baseline description

Categorical variables were expressed as counts and percentages, while continuous variables were presented as medians.

#### 2.4.2 Disproportionality analysis

In the study, disproportionality analyses were conducted using two common algorithms: the Reporting Odds Ratio (ROR) and the Proportional Reporting Ratio (PRR). Details of the two methods are provided in the two-by-two contingency tables (Supplementary Table 1). A positive signal was defined as either (i) the lower bound of the 95% confidence interval (CI) for ROR > 1 and *N* ≥3, or (ii) PRR ≥2, χ^2^ ≥ 4, and *N* ≥3 (Supplementary Table 2). Both ROR and PRR rely on the disproportional distribution of spontaneous reports and are used to screen for potential associations between vaccine and AE. The ROR measures the relative disproportionality of reporting for a specific vaccine-event pair and is generally more sensitive for early signal detection, whereas the PRR compares proportional differences and offers higher specificity in high-frequency or background-fluctuating events. This methodological framework is widely used in VAERS-based studies and pharmacovigilance signal detection (21, 22).

#### 2.4.3 Stratified analysis

Counts, proportions, and signal metrics for each PT were reported at both overall and severity-specific levels (serious vs. non-serious events). Stratified analyses were performed by age (< 18, 18–64, and ≥65 years), sex, vaccine manufacturer (Janssen, Moderna, and Pfizer-BioNTech), and vaccine valency (monovalent and bivalent). These age groups were selected to represent commonly used demographic categories in vaccine safety surveillance: children/adolescents, adults, and older adults. In addition, a sensitivity analysis stratified by age < 40 and ≥40 years was performed.

## 3 Results

### 3.1 Baseline characteristics

A total of 52,518 reports following COVID-19 vaccination were identified, comprising 59,015 AEs between December 2020 and January 2025. As shown in Table 1, arrhythmia-related AEs were most frequently observed in adults aged 18–64 years (44.6%), followed by older adults aged ≥65 years (24.8%) and adolescents aged < 18 years (1.7%). Females and males accounted for 59.4% and 38.9%, respectively. The median time from vaccination to symptom onset was 1 day.

**Table 1:** Baseline characteristics.

Characteristics	Total	< 18	18–64	≥65
Number	52,518	909	23,424	10,804
Report number	59,015	1,010	25,970	12,686
Sex				
Male	20,448 (38.9%)	444 (48.8%)	7,821 (33.4%)	5,208 (48.2%)
Female	31,190 (59.4%)	461 (50.7%)	15,409 (65.8%)	5,515 (51.0%)
Unknown	880 (1.7%)	4 (0.4%)	194 (0.8%)	81 (0.7%)
Median onset interval, days	1	1	0	9
Vaccine administered alone, *n* (%)	51,521 (98.1%)	890 (97.9%)	23,021 (98.3%)	10,470 (96.9%)
Serious outcome, *n* (%)	24,527 (46.7%)	430 (47.3%)	8,436 (36.0%)	7,207 (66.7%)
Death, *n* (%)	4,889 (9.3%)	43 (4.7%)	1,053 (4.5%)	2,514 (23.3%)

The vast majority of cases (98.1%) involved only COVID-19 vaccination without co-administration of other vaccines. Overall, 46.7% of events were classified as serious adverse events, and 9.3% of reports involved death. The proportions of serious and fatal events were higher among older adults (66.7% and 23.3%) than among adults (36.0% and 4.5%), respectively.

### 3.2 Disproportionality analysis

**Figure 1:**
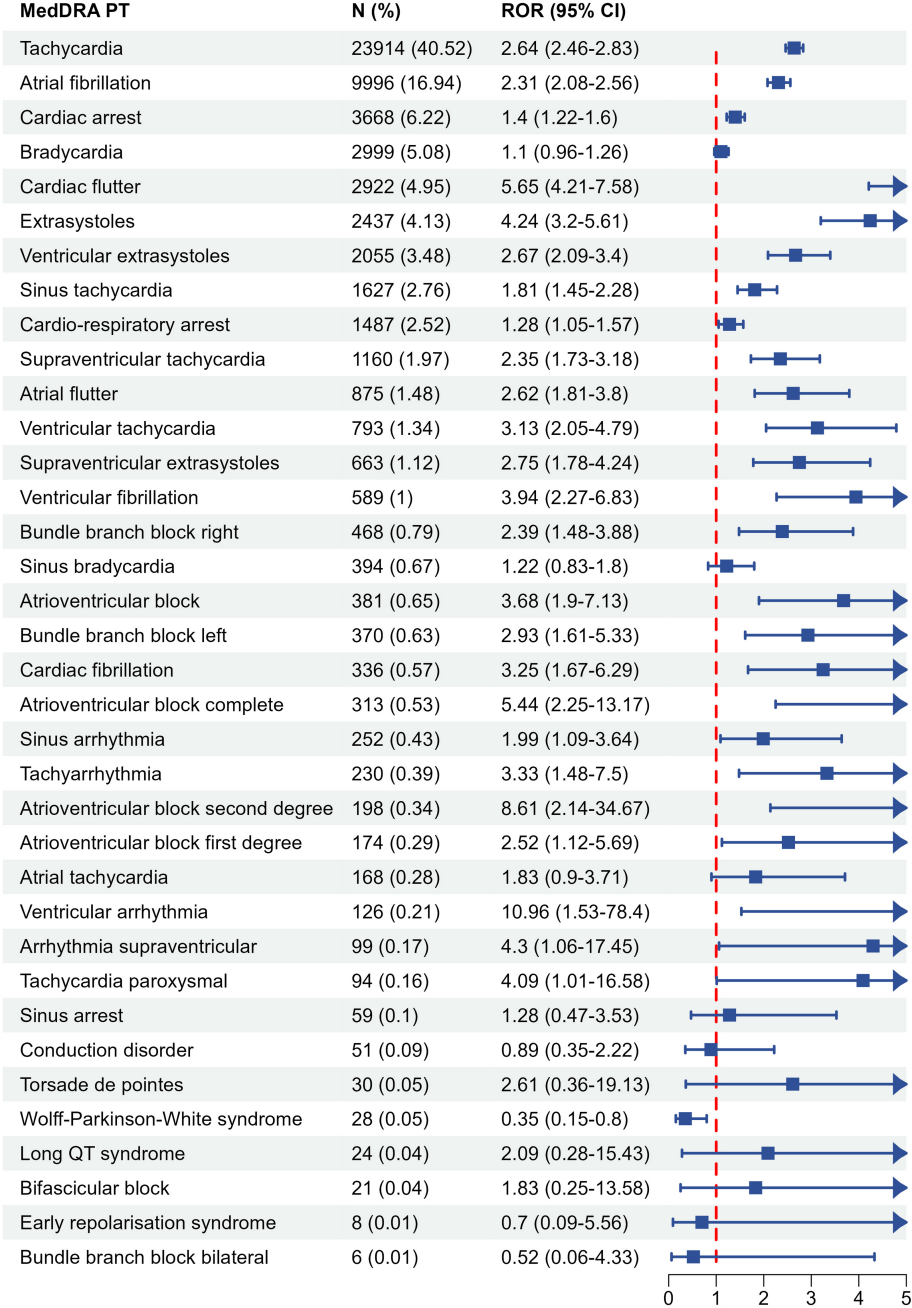
Disproportionality analysis of arrhythmia-related AEs following COVID-19 vaccination.

Disproportionality analysis revealed multiple MedDRA Preferred Terms (PTs) with significant reporting signals (Figure 1, Supplementary Table 3). Tachycardia (40.5%) was the most commonly reported event, showing the strongest signal (ROR = 2.64, 95% CI 2.46–2.83; PRR = 2.63, χ^2^ = 776.5), followed by atrial fibrillation (16.9%) with similarly significant signal strength (ROR = 2.31, 95% CI 2.08–2.56; PRR = 2.31, χ^2^ = 269). Additionally, cardiac arrest and bradycardia were uncommon, accounting for 6.2% and 5.1% of all AEs, respectively. Among supraventricular arrhythmias, atrial flutter (ROR = 5.65, 95% CI 4.21–7.58) and extrasystoles (ROR = 4.24, 95% CI 3.20–5.61) exhibited the strongest signals. Ventricular arrhythmias also showed elevated signals, including ventricular tachycardia (ROR = 3.13, 95% CI 2.05–4.79), ventricular fibrillation (ROR = 3.94, 95% CI 2.27–6.83), and ventricular arrhythmia (ROR = 10.96, 95% CI 1.53–78.40). Certain conduction abnormalities demonstrated significant disproportionality as well, particularly atrioventricular (AV) block (ROR = 3.68) and complete AV block (ROR = 5.44, 95% CI 2.25–13.17). Overall, tachycardia and atrial fibrillation represented the most frequently reported events, while atrial flutter, extrasystoles, and AV block showed the strongest signal strengths.

### 3.3 Stratified analyses

#### 3.3.1 Serious and non-serious AEs

Among all cases, 24,527 (46.7%) were classified as serious and 27,991 (53.3%) as non-serious (Table 2). In the serious group, life-threatening arrhythmias predominated, including cardiac arrest (12.4%), cardio-respiratory arrest (5.1%), ventricular fibrillation (1.9%), and AV block (1.0%). AV block (ROR = 10.06, 95% CI 2.50–40.43) and second-degree AV block (ROR = 10.24, 95% CI 1.43–73.18) displayed the highest signal strength. In contrast, the non-serious group was dominated by common supraventricular arrhythmias, including tachycardia (49.5%), atrial fibrillation (13.3%), atrial flutter (8.3%), and extrasystoles (5.7%). Atrial flutter (ROR = 6.09, 95% CI 4.44–8.35), extrasystoles (ROR = 3.94, 95% CI 2.89–5.37), and supraventricular tachycardia (ROR = 3.88, 95% CI 2.28–6.59) demonstrated the strongest signals.

**Table 2:** Disproportionality analysis of serious and non-serious arrhythmia-related AEs by MedDRA PTs.

MedDRA PT	Serious (28,460)			Non-serious (30,555)		
	*N* (%)	ROR (95% Cl)	PRR	*N* (%)	ROR (95% Cl)	PRR
Tachycardia	8,785 (30.87)	1.87 (1.67–2.08)	1.86 (129.84)	15,129 (49.51)	3.11 (2.83–3.41)	3.1 (630.36)
Atrial fibrillation	5,948 (20.9)	2.86 (2.43–3.37)	2.86 (174.8)	4,048 (13.25)	1.65 (1.44–1.88)	1.65 (55.33)
Cardiac arrest	3,519 (12.36)	1.27 (1.1–1.47)	1.27 (10.72)	149 (0.49)	0.45 (0.3–0.66)	0.45 (17.43)
Cardio-respiratory arrest	1,456 (5.12)	1.28 (1.02–1.6)	1.28 (4.64)	31 (0.1)	0.14 (0.08–0.25)	0.14 (61.55)
Bradycardia	1,198 (4.21)	0.91 (0.73–1.12)	0.91 (0.85)	1,801 (5.89)	1.17 (0.99–1.39)	1.17 (3.44)
Sinus tachycardia	868 (3.05)	1.14 (0.87–1.5)	1.14 (0.91)	759 (2.48)	2.95 (1.96–4.43)	2.95 (29.98)
Extrasystoles	705 (2.48)	5.57 (2.89–10.75)	5.57 (33.32)	1732 (5.67)	3.94 (2.89–5.37)	3.94 (87.87)
Ventricular extrasystoles	637 (2.24)	1.81 (1.21–2.7)	1.81 (8.75)	1418 (4.64)	3.15 (2.32–4.28)	3.15 (59.82)
Ventricular tachycardia	624 (2.19)	2.77 (1.69–4.56)	2.77 (17.69)	169 (0.55)	2.63 (1.16–5.93)	2.63 (5.84)
Atrial flutter	580 (2.04)	2.06 (1.32–3.22)	2.06 (10.57)	295 (0.97)	3.06 (1.57–5.93)	3.06 (12.09)
Supraventricular tachycardia	578 (2.03)	1.42 (0.98–2.06)	1.42 (3.39)	582 (1.9)	3.88 (2.28–6.59)	3.88 (29.19)
Ventricular fibrillation	547 (1.92)	2.99 (1.73–5.19)	2.99 (16.84)	42 (0.14)	–	–
Cardiac flutter	377 (1.32)	4.47 (1.99–10.01)	4.47 (15.9)	2,545 (8.33)	6.09 (4.44–8.35)	6.09 (163.32)
Bundle branch block right	300 (1.05)	2.13 (1.14–4.01)	2.13 (5.82)	168 (0.55)	2.24 (1.05–4.77)	2.24 (4.6)
Atrioventricular block complete	291 (1.02)	5.17 (1.93–13.88)	5.17 (13.28)	22 (0.07)	2.05 (0.28–15.22)	2.05 (0.52)
Atrioventricular block	283 (0.99)	10.06 (2.5–40.43)	10.06 (16.21)	98 (0.32)	1.31 (0.61–2.81)	1.31 (0.47)
Bundle branch block left	264 (0.93)	1.88 (1–3.53)	1.88 (3.95)	106 (0.35)	9.89 (1.38–70.83)	9.89 (7.91)
Sinus bradycardia	218 (0.77)	0.97 (0.58–1.61)	0.97 (0.02)	176 (0.58)	1.37 (0.76–2.45)	1.37 (1.11)
Supraventricular extrasystoles	200 (0.7)	1.42 (0.75–2.68)	1.42 (1.19)	463 (1.52)	3.93 (2.16–7.14)	3.93 (23.43)
Cardiac fibrillation	151 (0.53)	3.58 (1.14–11.22)	3.58 (5.47)	185 (0.61)	2.88 (1.28–6.48)	2.88 (7.11)
Atrioventricular block second degree	144 (0.51)	10.24 (1.43–73.18)	10.24 (8.28)	54 (0.18)	5.04 (0.7–36.4)	5.04 (3.18)
Tachyarrhythmia	127 (0.45)	3.01 (0.96–9.46)	3.01 (3.93)	103 (0.34)	3.2 (1.02–10.09)	3.2 (4.41)
Sinus arrhythmia	119 (0.42)	1.21 (0.56–2.59)	1.21 (0.24)	133 (0.44)	3.1 (1.15–8.38)	3.1 (5.53)
Atrioventricular block first degree	119 (0.42)	1.69 (0.69–4.14)	1.69 (1.36)	55 (0.18)	5.13 (0.71–37.07)	5.13 (3.26)
Atrial tachycardia	87 (0.31)	1.55 (0.57–4.21)	1.55 (0.74)	81 (0.27)	1.89 (0.69–5.15)	1.89 (1.59)
Ventricular arrhythmia	75 (0.26)	–	–	51 (0.17)	4.76 (0.66–34.42)	4.76 (2.91)
Arrhythmia supraventricular	50 (0.18)	–	–	49 (0.16)	2.28 (0.56–9.4)	2.28 (1.39)
Sinus arrest	46 (0.16)	1.64 (0.4–6.74)	1.64 (0.47)	13 (0.04)	0.61 (0.14–2.69)	0.61 (0.44)
Tachycardia paroxysmal	44 (0.15)	3.13 (0.43–22.71)	3.13 (1.42)	50 (0.16)	4.66 (0.64–33.76)	4.66 (2.82)
Conduction disorder	34 (0.12)	0.81 (0.25–2.62)	0.81 (0.13)	17 (0.06)	0.79 (0.18–3.43)	0.79 (0.1)
Torsade de pointes	29 (0.1)	2.06 (0.28–15.14)	2.06 (0.53)	1 (0)	–	–
Bifascicular block	18 (0.06)	1.28 (0.17–9.59)	1.28 (0.06)	3 (0.01)	–	–
Long QT syndrome	17 (0.06)	–	–	7 (0.02)	0.65 (0.08–5.31)	0.65 (0.16)
Wolff-Parkinson-White syndrome	14 (0.05)	0.25 (0.08–0.76)	0.25 (7.05)	14 (0.05)	0.44 (0.13–1.51)	0.44 (1.81)
Early repolarisation syndrome	5 (0.02)	0.36 (0.04–3.04)	0.36 (0.97)	3 (0.01)	–	–
Bundle branch block bilateral	3 (0.01)	–	–	3 (0.01)	0.28 (0.03–2.69)	0.28 (1.39)

#### 3.3.2 Age

In individuals aged < 18 years, arrhythmia signals were primarily driven by tachycardia (ROR = 1.95, 95% CI 1.63–2.33) and atrial flutter (ROR = 26.70, 95% CI 3.67–194.23) (Supplementary Table 4). Among adults aged 18–64 years, reports were mainly associated with tachycardia (ROR = 2.30, 95% CI 2.05–2.57) and atrial fibrillation (ROR = 2.23, 95% CI 1.71–2.92), followed by atrial flutter (ROR = 4.56) and ventricular fibrillation (ROR = 3.07) (Supplementary Table 4). In older adults aged ≥ 65 years, overall signal strength was relatively lower, but arrhythmia-related AEs were more concentrated in ventricular arrhythmias and conduction abnormalities (Supplementary Table 4). Atrial fibrillation (ROR = 1.58) remained the most frequent event, while ventricular arrhythmias (ROR >2) and atrioventricular or bundle branch blocks showed more prominent disproportionality than in younger groups.

In an additional age-specific sensitivity analysis using a cutoff of 40 years, the main disproportionality patterns were largely consistent with the primary analysis (Supplementary Table 5). Tachycardia remained the most frequently reported PT in both the < 40-year and ≥40-year groups. Positive signals for cardiac flutter, sinus tachycardia, extrasystoles, ventricular extrasystoles, supraventricular tachycardia, and ventricular tachycardia were observed in both age strata. However, some age-related heterogeneity was noted: atrial fibrillation, bradycardia, cardiac arrest, cardio-respiratory arrest, ventricular fibrillation, and several conduction-related events were more prominent among individuals aged ≥40 years.

#### 3.3.3 Sex

Differences in the spectrum of adverse events were also observed by sex (Supplementary Table 6). Among females, the leading AEs were tachycardia (48.5%), atrial fibrillation (13.6%), and atrial flutter (5.6%), all showing significant positive signals (ROR = 2.57, 2.31, 5.97, respectively). Among males, the most common events were tachycardia (28.7%), atrial fibrillation (21.9%), and cardiac arrest (9.2%). The overall trend indicated that females were more frequently associated with supraventricular arrhythmias, whereas males exhibited higher proportions of ventricular and conduction abnormalities.

#### 3.3.4 Vaccine type

AEs also varied across vaccine platforms (Supplementary Table 7). For mRNA vaccines, the predominant events were tachycardia and atrial fibrillation. The Moderna subgroup showed relatively stronger signals for extrasystoles and atrial flutter, whereas the Pfizer–BioNTech subgroup was characterized mainly by supraventricular arrhythmias. In contrast, arrhythmia-related AEs reported after the adenoviral vector vaccine were fewer. In the Janssen subgroup, cardiac arrest and bradyarrhythmia-related events showed relatively higher RORs than several tachyarrhythmic events, but these estimates were not statistically robust. Therefore, Janssen-specific findings, particularly for bradyarrhythmias, should be interpreted cautiously and should not be considered evidence of a specific association with the adenoviral vector vaccine.

## 4 Discussion

This study systematically analyzed arrhythmia-related AEs reported after COVID-19 vaccination between December 2020 and January 2025 using data from VAERS. The findings revealed that AE reports were predominantly concentrated in the early post-vaccination period, with tachycardia and atrial fibrillation being the most frequently reported types. Meanwhile, atrial flutter, extrasystoles, and AV block showed the strongest signal intensities. Stratified analyses indicated that severe AEs were mainly characterized by ventricular and conduction abnormalities, whereas non-serious AEs were primarily supraventricular in nature. Notably, significant variations across age, sex, and vaccine platforms suggest that the risk of vaccine-associated arrhythmias may be shaped by the interaction between individual characteristics and platform-specific mechanisms.

The median onset time of arrhythmia after COVID-19 vaccination was 1 day, exhibiting a distinct early-onset pattern. This finding aligned with a nationwide retrospective study in South Korea (HR = 3.56, 95% CI 1.15–11.04) and a self-controlled case series in the United Kingdom within 1–7 days after the second mRNA-1273 dose (RR = 1.93, 95% CI 1.25–2.96) (15, 23). Together, these data support a temporal relationship between vaccination and the reported arrhythmia-related events, suggesting that the immediate post-vaccination period may represent a clinically relevant window for the reporting and monitoring of these events. However, this temporal association should not be interpreted as direct evidence of causality. This is particularly important for tachycardia, the most frequently reported arrhythmia-related AE in our study, because tachycardia after vaccination may clinically overlap with Long COVID, post-viral dysautonomia, and postural orthostatic tachycardia syndrome (POTS)-like presentations, especially among individuals with prior SARS-CoV-2 infection (24). Previous studies have suggested that COVID-19 may precipitate autonomic dysfunction, with symptoms such as palpitations, persistent tachycardia, dizziness, fatigue, exercise intolerance, and orthostatic intolerance (25). Potential mechanisms include endothelial injury, persistent inflammation, autonomic nervous system involvement, physical deconditioning, and heightened sympathetic activation, all of which may contribute to abnormal heart-rate regulation (24, 25). Because VAERS does not consistently provide verified information on prior SARS-CoV-2 infection, Long COVID status, orthostatic vital signs, autonomic testing, or detailed clinical adjudication, we could not distinguish vaccine-triggered tachycardia from post-COVID dysautonomia or other background conditions. Therefore, although the early-onset pattern is clinically relevant, tachycardia and other arrhythmia-related events in this study should be interpreted as pharmacovigilance reporting signals rather than definitive evidence of vaccine-induced arrhythmia.

Previous research indicates that both mRNA and adenoviral vector vaccines can induce transient systemic immune activation. For mRNA vaccines, cytokine responses characterized by IL-15, IFN-γ, and CXCL10/IP-10 emerge within 24 hours post-vaccination and extend to IL-6 and TNF-α after the second dose, reflecting a rapid yet transient inflammatory peak (26). Adenoviral vector vaccines also trigger short-lived activation of platelet, coagulation, and innate immune pathways, typically resolving within about seven days (27). Under this inflammatory milieu, proinflammatory cytokines such as IL-6, TNF-α, and IL-1β can modulate cardiac ion channels, prolonging action potential duration or suppressing repolarizing potassium currents (IKs), thereby altering myocardial repolarization properties. Notably, such electrophysiological disturbances may occur even in the absence of overt structural myocardial injury, leading to transient electrical instability and arrhythmias (28). The cumulative effects of multiple cytokines during systemic inflammation may further increase susceptibility to rhythm disturbances, providing a plausible biological basis for the early-onset pattern observed in vaccine-associated arrhythmias.

Distinct arrhythmic profiles were observed between vaccine platforms. mRNA vaccine reports were predominantly characterized by tachyarrhythmia-related events such as atrial fibrillation and ventricular tachycardia, whereas adenoviral vector vaccine-related reports showed fewer events and required cautious interpretation for bradyarrhythmia- and conduction-related findings. This divergence likely reflects fundamental differences in immune activation mechanisms. mRNA vaccines activate endosomal TLR7/8 and cytosolic MDA5 pathways, inducing a transient release of type I interferons and proinflammatory mediators including IL-6 and TNF-α (29). These cytokines may disrupt autonomic balance and alter myocardial ion channel function, resulting in prolonged action potentials, increased repolarization dispersion, and triggered depolarizations—mechanisms that predispose to atrial fibrillation, ventricular tachycardia, and torsades de pointes (30). In contrast, adenoviral vector vaccines may induce mild endothelial inflammation and coagulation activation, leading to localized perfusion disturbances or conduction system involvement, thereby potentially contributing to bradyarrhythmia- or conduction-related reporting patterns (27).

This study also demonstrated that severe events were more common among older adults and males, whereas supraventricular arrhythmias predominated among females. Prior evidence indicates that sex- and age-related differences in electrophysiology, ion channel expression, and autonomic regulation may shape the cardiac response to inflammatory or immunologic stress. Males tend to exhibit higher sympathetic activity and lower vagal tone and are more prone to cytokine-driven (e.g., IL-6, TNF-α) action potential and QTc prolongation, increasing the risk of severe arrhythmias. In contrast, females display greater vagal predominance and shorter AV nodal conduction times, predisposing to supraventricular arrhythmias while conferring relative resistance to ventricular arrhythmias under ischemic or inflammatory stress (30, 31). Collectively, these findings underscore the role of demographic and biological factors in shaping vaccine-associated arrhythmia risks and highlight the importance of incorporating individualized risk assessment and monitoring strategies.

Although the overall incidence of vaccine-related arrhythmias remains low, even marginal increases could have public health implications given the vast global vaccination base. This study suggests that arrhythmias tend to occur shortly after vaccination, with distinct stratification by type and severity. Enhanced early post-vaccination signal monitoring and symptom-triggered ECG evaluation may facilitate timely detection of potential rhythm disturbances. Future pharmacovigilance should adopt stratified risk communication and active surveillance approaches tailored to vaccine platform and population characteristics to improve the precision of safety monitoring.

From a clinical and public health perspective, our findings may help inform adverse event monitoring following immunization. The median onset interval of 1 day suggests that the immediate post-vaccination period may represent an important window for the recognition and assessment of arrhythmia-related events, and may be more relevant than delayed follow-up for early symptom monitoring. Individuals who develop palpitations, persistent tachycardia, syncope, presyncope, chest discomfort, or dyspnea shortly after vaccination may warrant timely clinical evaluation, including electrocardiography when clinically indicated. In addition, although atrial flutter and atrioventricular block were less frequently reported than tachycardia, they showed strong disproportionality signals and are clinically more serious rhythm or conduction disturbances. Therefore, post-vaccination safety surveillance should not focus solely on isolated tachycardia, but should also consider potentially serious arrhythmic or conduction events, particularly among older individuals, those with pre-existing cardiovascular disease, or those presenting with suggestive symptoms. However, given the passive reporting nature of VAERS and the inability to infer true incidence or causality, these findings support risk-informed and symptom-triggered monitoring rather than routine electrocardiographic screening for all vaccine recipients.

The present study offers several strengths. The long observation period (2020–2024) and large sample size allow for comprehensive assessment of safety trends throughout vaccine rollout. The use of both ROR and PRR methods enhances the robustness of signal detection from complementary statistical perspectives. Furthermore, subgroup analyses by age, sex, and vaccine type reveal population- and platform-specific risk differences, strengthening clinical and public health interpretation.

Nevertheless, several limitations must be acknowledged. As a spontaneous reporting system, VAERS is subject to both underreporting and overreporting. The former may result from mild events or reporting noncompliance, whereas the latter may be influenced by public awareness or media attention (32, 33). Incomplete clinical data, such as underlying conditions, concomitant medications, prior arrhythmia history, prior SARS-CoV-2 infection, Long COVID status, and healthcare-seeking or reporting behavior, limit control of confounding factors. Although stratified analyses by age, sex, vaccine manufacturer, and vaccine valency were performed, residual confounding from demographic factors, vaccine-related factors, and unmeasured clinical characteristics cannot be excluded, as these variables are not consistently available or verifiable in VAERS. ROR and PRR reflect relative reporting tendencies rather than true incidence and thus cannot infer causality directly. Despite these limitations, large-scale cross-platform analyses such as this provide valuable insights for early signal detection and hypothesis generation.

Future studies should leverage active surveillance systems such as the Vaccine Safety Datalink (VSD) (34) and prospective designs to verify causal associations between different vaccine platforms and arrhythmia risk. Integration of electronic health records and claims data could further elucidate time–dose relationships between immune activation and electrophysiologic imbalance. Additionally, developing individualized risk prediction models and dynamic monitoring strategies for high-risk populations, alongside promoting international data sharing and standardized signal analysis, will be essential to achieve comprehensive, lifecycle evaluation of vaccine safety.

## 5 Conclusion

Cardiac arrhythmias following COVID-19 vaccination typically occurred within the early post-vaccination period and demonstrated clear platform- and population-specific differences. Although the overall risk is low, enhanced early monitoring and stratified pharmacovigilance are warranted to optimize vaccine safety.

## Data Availability

Publicly available datasets were analyzed in this study. This data can be found here: https://vaers.hhs.gov/.
