# Supplementary material for: Cardiac arrhythmias following COVID-19 vaccination: insights from the U.S. vaccine adverse event reporting system (VAERS), December 2020—January 2025, United States
Source: Front Public Health. 2026 Jun 8;14:1762082. doi: 10.3389/fpubh.2026.1762082 (PMC13284064; doi:10.3389/fpubh.2026.1762082)
Supplement: Supplementary file 1 [file Data_Sheet_1.docx]

**Supplementary Table 1.** Two-by-two contingency table for disproportionality analyses.

|  | Target AEs | Other AEs | Total |
| --- | --- | --- | --- |
| Covid-19 vaccines | a | b | a+b |
| Other vaccines | c | d | c+d |
| Total | a+c | b+d | a+b+c+d |

AEs, adverse events; a, number of reports containing both the target vaccine and target adverse event; b, number of reports containing other adverse events for the target vaccine; c, number of reports containing the target adverse event for other vaccines; d, number of reports containing other vaccines and other adverse events. Other vaccines were defined as vaccine records in VAERS during the study period that were not classified as the five prespecified target COVID-19 vaccine products. Details of these vaccines are provided in **Supplementary Table 8**.

**Supplementary Table 2.** Two major algorithms used for signal detection.

| Algorithms | Equation | Criteria |
| --- | --- | --- |
| ROR | ROR=ad/b/c | lower limit of 95% CI>1, N≥3 |
|  | 95%CI=eln(ROR)±1.96(1/a+1/b+1/c+1/d)^0.5 |  |
| PRR | PRR=a(c+d)/c/(a+b) | PRR≥2, χ2≥4, N≥3 |
|  | χ2=[(ad-bc)^2](a+b+c+d)/[(a+b)(c+d)(a+c)(b+d)] |  |

a, number of reports containing both the target drug and target adverse drug reaction; b, number of reports containing other adverse drug reaction of the target drug; c, number of reports containing the target adverse drug reaction of other drugs; d, number of reports containing other drugs and other adverse drug reactions. 95%CI, 95% confidence interval; N, the number of reports; χ2, chi-squared.

**Supplementary Table 3.** MedDRA PTs with significant disproportionality signals for arrhythmia-related AEs following COVID-19 vaccination.

| MedDRA PT | N (%) | ROR (95%Cl) | PRR |
| --- | --- | --- | --- |
| Tachycardia | 23914 (40.52) | 2.64 (2.46 - 2.83) | 2.63 (776.51) |
| Atrial fibrillation | 9996 (16.94) | 2.31 (2.08 - 2.56) | 2.31 (269) |
| Cardiac arrest | 3668 (6.22) | 1.4 (1.22 - 1.6) | 1.4 (24.44) |
| Bradycardia | 2999 (5.08) | 1.1 (0.96 - 1.26) | 1.1 (2.01) |
| Cardiac flutter | 2922 (4.95) | 5.65 (4.21 - 7.58) | 5.65 (169.53) |
| Extrasystoles | 2437 (4.13) | 4.24 (3.2 - 5.61) | 4.24 (121.28) |
| Ventricular extrasystoles | 2055 (3.48) | 2.67 (2.09 - 3.4) | 2.67 (67.64) |
| Sinus tachycardia | 1627 (2.76) | 1.81 (1.45 - 2.28) | 1.81 (27.19) |
| Cardio-respiratory arrest | 1487 (2.52) | 1.28 (1.05 - 1.57) | 1.28 (5.81) |
| Supraventricular tachycardia | 1160 (1.97) | 2.35 (1.73 - 3.18) | 2.35 (32.02) |
| Atrial flutter | 875 (1.48) | 2.62 (1.81 - 3.8) | 2.62 (28.21) |
| Ventricular tachycardia | 793 (1.34) | 3.13 (2.05 - 4.79) | 3.13 (31.12) |
| Supraventricular extrasystoles | 663 (1.12) | 2.75 (1.78 - 4.24) | 2.75 (22.59) |
| Ventricular fibrillation | 589 (1) | 3.94 (2.27 - 6.83) | 3.94 (27.91) |
| Bundle branch block right | 468 (0.79) | 2.39 (1.48 - 3.88) | 2.39 (13.32) |
| Sinus bradycardia | 394 (0.67) | 1.22 (0.83 - 1.8) | 1.22 (1.07) |
| Atrioventricular block | 381 (0.65) | 3.68 (1.9 - 7.13) | 3.68 (17.17) |
| Bundle branch block left | 370 (0.63) | 2.93 (1.61 - 5.33) | 2.93 (13.54) |
| Cardiac fibrillation | 336 (0.57) | 3.25 (1.67 - 6.29) | 3.25 (13.63) |
| Atrioventricular block complete | 313 (0.53) | 5.44 (2.25 - 13.17) | 5.44 (17.85) |
| Sinus arrhythmia | 252 (0.43) | 1.99 (1.09 - 3.64) | 1.99 (5.21) |
| Tachyarrhythmia | 230 (0.39) | 3.33 (1.48 - 7.5) | 3.33 (9.55) |
| Atrioventricular block second degree | 198 (0.34) | 8.61 (2.14 - 34.67) | 8.61 (13.32) |
| Atrioventricular block first degree | 174 (0.29) | 2.52 (1.12 - 5.69) | 2.52 (5.33) |
| Atrial tachycardia | 168 (0.28) | 1.83 (0.9 - 3.71) | 1.83 (2.85) |
| Ventricular arrhythmia | 126 (0.21) | 10.96 (1.53 - 78.4) | 10.96 (8.98) |
| Arrhythmia supraventricular | 99 (0.17) | 4.3 (1.06 - 17.45) | 4.3 (4.97) |
| Tachycardia paroxysmal | 94 (0.16) | 4.09 (1.01 - 16.58) | 4.09 (4.57) |
| Sinus arrest | 59 (0.1) | 1.28 (0.47 - 3.53) | 1.28 (0.23) |
| Conduction disorder | 51 (0.09) | 0.89 (0.35 - 2.22) | 0.89 (0.07) |
| Torsade de pointes | 30 (0.05) | 2.61 (0.36 - 19.13) | 2.61 (0.96) |
| Wolff-Parkinson-White syndrome | 28 (0.05) | 0.35 (0.15 - 0.8) | 0.35 (6.85) |
| Long QT syndrome | 24 (0.04) | 2.09 (0.28 - 15.43) | 2.09 (0.54) |
| Bifascicular block | 21 (0.04) | 1.83 (0.25 - 13.58) | 1.83 (0.36) |
| Early repolarisation syndrome | 8 (0.01) | 0.7 (0.09 - 5.56) | 0.7 (0.12) |
| Bundle branch block bilateral | 6 (0.01) | 0.52 (0.06 - 4.33) | 0.52 (0.38) |

**Supplementary Table 4.** Disproportionality analysis of arrhythmia-related AEs by age group.

| MedDRA PT | <18 (909) | | |  | 18-64 (23424) | | |  | ≥65 (10804) | | |
| --- | --- | --- | --- | --- | --- | --- | --- | --- | --- | --- | --- |
|  | N (%) | ROR (95%Cl) | PRR |  | N (%) | ROR (95%Cl) | PRR |  | N (%) | ROR (95%CI) | PRR |
| Tachycardia | 475 (47.03) | 1.95 (1.63-2.33) | 1.94 (55.81) |  | 13119 (50.52) | 2.3 (2.05-2.57) | 2.29 (219.34) |  | 2182 (17.2) | 1.69 (1.39-2.05) | 1.69 (28.75) |
| Bradycardia | 113 (11.19) | 0.85 (0.64-1.12) | 0.85 (1.37) |  | 1354 (5.21) | 1.58 (1.18-2.12) | 1.58 (9.45) |  | 760 (5.99) | 3.94 (2.4-6.46) | 3.94 (34.33) |
| Sinus tachycardia | 73 (7.23) | 2.12 (1.33-3.39) | 2.12 (10.33) |  | 687 (2.64) | 2.23 (1.46-3.39) | 2.23 (13.34) |  | 172 (1.36) | 2.85 (1.17-6.94) | 2.85 (5.84) |
| Cardiac arrest | 45 (4.46) | 0.47 (0.32-0.69) | 0.47 (15.87) |  | 476 (1.83) | 1.25 (0.9-1.73) | 1.25 (3.96) |  | 1375 (10.84) | 2 (1.53-2.61) | 2 (27.34) |
| Cardiac flutter | 40 (3.96) | 26.7 (3.67-194.23) | 26.7 (24.13) |  | 1673 (6.44) | 4.08 (2.68-6.22) | 4.08 (50.5) |  | 307 (2.42) | 1.96 (1.12-3.41) | 1.96 (5.83) |
| Ventricular extrasystoles | 33 (3.27) | 5.51 (1.95-15.54) | 5.51 (13.16) |  | 1101 (4.24) | 2.36 (1.59-3.51) | 2.36 (19.22) |  | 395 (3.11) | 1.49 (0.97-2.29) | 1.49 (3.33) |
| Sinus arrhythmia | 33 (3.27) | 7.34 (2.25-23.94) | 7.34 (15.06) |  | 387 (1.49) | 2.63 (1.69-4.09) | 2.63 (20.02) |  | 20 (0.16) | - | - |
| Ventricular tachycardia | 30 (2.97) | 5.01 (1.76-14.21) | 5.01 (11.31) |  | 670 (2.58) | 2.75 (1.84-4.09) | 2.75 (27.22) |  | 264 (2.08) | 4.38 (1.81-10.6) | 4.37 (12.78) |
| Sinus bradycardia | 23 (2.28) | 15.35 (2.07-113.68) | 15.35 (12.86) |  | 537 (2.06) | 2.14 (1.33-3.43) | 2.14 (10.92) |  | 113 (0.89) | 1.56 (0.69-3.55) | 1.56 (1.15) |
| Supraventricular tachycardia | 21 (2.08) | 1 (0.51-1.97) | 1 (0) |  | 679 (2.61) | 2.52 (1.67-3.8) | 2.52 (21.24) |  | 221 (1.74) | 2.62 (1.23-5.55) | 2.62 (6.77) |
| Extrasystoles | 20 (1.98) | 4.45 (1.32-14.97) | 4.45 (6.98) |  | 931 (3.59) | 2.67 (1.8-3.94) | 2.67 (24.64) |  | 276 (2.18) | 1.91 (1.07-3.4) | 1.91 (4.95) |
| Cardio-respiratory arrest | 18 (1.78) | 0.31 (0.18-0.54) | 0.31 (19.15) |  | 423 (1.63) | 1.15 (0.82-1.61) | 1.15 (2.43) |  | 724 (5.71) | 2.86 (1.85-4.41) | 2.86 (24.63) |
| Bundle branch block right | 15 (1.49) | 10.01 (1.32-75.79) | 10.01 (7.6) |  | 374 (1.44) | 2.47 (1.59-3.83) | 2.47 (15.96) |  | 126 (0.99) | 2.09 (0.85-5.1) | 2.09 (2.73) |
| Atrial fibrillation | 12 (1.19) | 2.67 (0.75-9.46) | 2.67 (2.51) |  | 2289 (8.81) | 2.23 (1.71-2.92) | 2.23 (36.58) |  | 4383 (34.55) | 2 (1.72-2.32) | 2 (87.02) |
| Atrioventricular block | 7 (0.69) | - | - |  | 460 (1.77) | 2.33 (1.56-3.47) | 2.33 (18.24) |  | 116 (0.91) | 2.4 (0.89-6.51) | 2.4 (3.17) |
| Atrioventricular block first degree | 6 (0.59) | 2 (0.4-9.92) | 2 (0.75) |  | 365 (1.4) | 2.23 (1.46-3.39) | 2.23 (12.93) |  | 87 (0.69) | 7.21 (1-51.75) | 7.21 (5.29) |
| Supraventricular extrasystoles | 5 (0.5) | 3.34 (0.39-28.56) | 3.34 (1.36) |  | 447 (1.72) | 2.95 (2.0-4.34) | 2.95 (32.24) |  | 146 (1.15) | 1.73 (0.81-3.69) | 1.73 (2.05) |
| Ventricular fibrillation | 5 (0.5) | 1.67 (0.32-8.6) | 1.67 (0.38) |  | 498 (1.91) | 2.22 (1.46-3.37) | 2.22 (12.93) |  | 158 (1.25) | - | - |
| Atrioventricular block complete | 5 (0.5) | - | - |  | 308 (1.18) | 2.73 (1.79-4.17) | 2.73 (23.76) |  | 109 (0.86) | 3.01 (0.96-9.48) | 3.01 (3.92) |
| Atrioventricular block second degree | 4 (0.4) | - | - |  | 330 (1.27) | 2.64 (1.72-4.03) | 2.64 (21.3) |  | 61 (0.48) | - | - |
| Ventricular arrhythmia | 4 (0.4) | - | - |  | 296 (1.14) | 2.62 (1.7-4.03) | 2.62 (20.9) |  | 15 (0.12) | - | - |
| Wolff-Parkinson-White syndrome | 4 (0.4) | 0.89 (0.2-3.98) | 0.89 (0.02) |  | 183 (0.7) | 2.05 (1.3-3.23) | 2.05 (9.04) |  | - | - | - |
| Tachyarrhythmia | 4 (0.4) | - | - |  | 283 (1.09) | 2.58 (1.69-3.93) | 2.58 (19.14) |  | 43 (0.34) | 3.56 (0.49-25.87) | 3.56 (1.8) |
| Conduction disorder | 3 (0.3) | 1 (0.17-5.99) | 1 (0) |  | 207 (0.8) | 2.14 (1.34-3.42) | 2.14 (10.9) |  | 12 (0.09) | - | - |
| Atrial tachycardia | 3 (0.3) | - | - |  | 277 (1.07) | 2.62 (1.7-4.03) | 2.62 (20.9) |  | 33 (0.26) | 0.68 (0.24-1.93) | 0.68 (0.52) |
| Early repolarisation syndrome | 2 (0.2) | 1.33 (0.12-14.72) | 1.33 (0.06) |  | 3 (0.01) | - | - |  | - | - | - |
| Long QT syndrome | 2 (0.2) | - | - |  | 189 (0.73) | 2.14 (1.35-3.4) | 2.14 (10.96) |  | 3 (0.02) | 0.25 (0.03-2.39) | 0.25 (1.7) |
| Atrial flutter | 1 (0.1) | - | - |  | 652 (2.51) | 2.43 (1.6-3.69) | 2.43 (17.94) |  | 342 (2.7) | 1.67 (1.02-2.71) | 1.67 (4.32) |
| Arrhythmia supraventricular | 1 (0.1) | - | - |  | 244 (0.94) | 2.43 (1.58-3.72) | 2.43 (16.72) |  | 19 (0.15) | 1.57 (0.21-11.76) | 1.57 (0.2) |
| Cardiac fibrillation | 1 (0.1) | - | - |  | - | - | - |  | 50 (0.39) | 2.07 (0.5-8.51) | 2.07 (1.07) |
| Bundle branch block left | 1 (0.1) | - | - |  | 253 (0.97) | 2.62 (1.69-4.05) | 2.62 (20.12) |  | 124 (0.98) | 2.57 (0.95-6.95) | 2.57 (3.71) |
| Torsade de pointes | 1 (0.1) | - | - |  | 197 (0.76) | 2.22 (1.44-3.42) | 2.22 (12.62) |  | 5 (0.04) | - | - |
| Bundle branch block bilateral | - | - | - |  | 6 (0.02) | - | - |  | 11 (0.09) | - | - |
| Tachycardia paroxysmal | - | - | - |  | 46 (0.18) | 2.47 (0.34 - 17.89) | 2.47 (0.85) |  | 9 (0.07) | - | - |
| Sinus arrest | - | - | - |  | 23 (0.09) | 1.23 (0.17 - 9.14) | 1.23 (0.04) |  | 23 (0.18) | 1.91 (0.26 - 14.11) | 1.91 (0.41) |
| Bifascicular block | - | - | - |  | 6 (0.02) | - | - |  | 11 (0.09) | - | - |

Supplementary Table 5. Sensitivity analysis of disproportionality signals for arrhythmia-related AEs stratified by age <40 and ≥40 years.

| MedDRA PT | <40 years | | | ≥40 years | | |
| --- | --- | --- | --- | --- | --- | --- |
|  | N  (%) | ROR  (95% CI) | PRR | N  (%) | ROR  (95% CI) | PRR |
| Tachycardia | 6695 (57.67) | 2.72 (2.43 - 3.04) | 2.71 (328.09) | 9081 (32.37) | 2.32 (2.05 - 2.63) | 2.32 (190.55) |
| Bradycardia | 803 (6.92) | 0.84 (0.7 - 1.02) | 0.84 (3.02) | 1424 (5.08) | 3.26 (2.26 - 4.71) | 3.26 (44.63) |
| Cardiac flutter | 736 (6.34) | 15.75 (7.05 - 35.18) | 15.75 (82.22) | 1284 (4.58) | 2.85 (1.98 - 4.09) | 2.84 (35.07) |
| Sinus tachycardia | 576 (4.96) | 2.18 (1.54 - 3.07) | 2.17 (20.38) | 577 (2.06) | 2.13 (1.33 - 3.41) | 2.13 (10.47) |
| Extrasystoles | 445 (3.83) | 5.19 (2.86 - 9.45) | 5.19 (36.35) | 928 (3.31) | 2.47 (1.66 - 3.67) | 2.47 (21.24) |
| Ventricular extrasystoles | 417 (3.59) | 5.35 (2.86 - 10.02) | 5.35 (34.58) | 1112 (3.96) | 1.8 (1.32 - 2.46) | 1.8 (14.13) |
| Atrial fibrillation | 308 (2.65) | 3.95 (2.11 - 7.42) | 3.95 (21.38) | 6376 (22.73) | 1.84 (1.62 - 2.1) | 1.84 (85.65) |
| Cardiac arrest | 281 (2.42) | 0.46 (0.36 - 0.59) | 0.46 (38.15) | 2192 (7.81) | 1.73 (1.4 - 2.16) | 1.73 (25.17) |
| Supraventricular tachycardia | 259 (2.23) | 1.85 (1.15 - 2.98) | 1.85 (6.54) | 593 (2.11) | 2.63 (1.57 - 4.39) | 2.63 (14.75) |
| Ventricular tachycardia | 125 (1.08) | 2.29 (1.07 - 4.91) | 2.29 (4.83) | 453 (1.61) | 4.3 (2.04 - 9.07) | 4.3 (17.47) |
| Sinus arrhythmia | 118 (1.02) | 2.16 (1.01 - 4.64) | 2.16 (4.14) | 53 (0.19) | 3.52 (0.49 - 25.47) | 3.52 (1.77) |
| Supraventricular extrasystoles | 117 (1.01) | 3.76 (1.39 - 10.17) | 3.75 (7.82) | 352 (1.25) | 1.95 (1.1 - 3.47) | 1.95 (5.37) |
| Bundle branch block right | 108 (0.93) | 2.77 (1.13 - 6.8) | 2.77 (5.42) | 225 (0.8) | 1.66 (0.85 - 3.23) | 1.66 (2.28) |
| Cardio-respiratory arrest | 106 (0.91) | 0.27 (0.19 - 0.38) | 0.27 (66.14) | 1022 (3.64) | 2 (1.42 - 2.81) | 2 (16.4) |
| Sinus bradycardia | 102 (0.88) | 1.31 (0.68 - 2.51) | 1.31 (0.67) | 188 (0.67) | 1.04 (0.58 - 1.87) | 1.04 (0.02) |
| Ventricular fibrillation | 73 (0.63) | 2.34 (0.86 - 6.41) | 2.34 (2.92) | 319 (1.14) | 10.6 (2.64 - 42.57) | 10.6 (17.28) |
| Atrial flutter | 42 (0.36) | Inf (NaN - Inf) | Inf (5.39) | 556 (1.98) | 1.85 (1.18 - 2.89) | 1.85 (7.51) |
| Atrioventricular block | 41 (0.35) | 5.26 (0.72 - 38.26) | 5.26 (3.37) | 207 (0.74) | 2.29 (1.02 - 5.16) | 2.29 (4.25) |
| Atrioventricular block second degree | 31 (0.27) | 3.98 (0.54 - 29.15) | 3.98 (2.16) | 102 (0.36) | Inf (NaN - Inf) | Inf (6.78) |
| Tachyarrhythmia | 28 (0.24) | 3.59 (0.49 - 26.42) | 3.59 (1.81) | 89 (0.32) | 2.96 (0.73 - 12.01) | 2.96 (2.53) |
| Atrial tachycardia | 27 (0.23) | 1.73 (0.41 - 7.29) | 1.73 (0.58) | 68 (0.24) | 1.13 (0.41 - 3.1) | 1.13 (0.06) |
| Atrioventricular block complete | 22 (0.19) | Inf (NaN - Inf) | Inf (2.82) | 180 (0.64) | 3.99 (1.27 - 12.48) | 3.99 (6.61) |
| Cardiac fibrillation | 22 (0.19) | Inf (NaN - Inf) | Inf (2.82) | 109 (0.39) | 3.62 (0.89 - 14.67) | 3.62 (3.73) |
| Atrioventricular block first degree | 21 (0.18) | 1.35 (0.32 - 5.75) | 1.35 (0.16) | 115 (0.41) | 7.64 (1.07 - 54.72) | 7.64 (5.72) |
| Bundle branch block left | 20 (0.17) | 1.28 (0.3 - 5.49) | 1.28 (0.11) | 222 (0.79) | 2.95 (1.22 - 7.16) | 2.95 (6.31) |
| Tachycardia paroxysmal | 18 (0.16) | Inf (NaN - Inf) | Inf (2.31) | 37 (0.13) | 2.46 (0.34 - 17.92) | 2.46 (0.84) |
| Ventricular arrhythmia | 16 (0.14) | Inf (NaN - Inf) | Inf (2.05) | 53 (0.19) | Inf (NaN - Inf) | Inf (3.52) |
| Conduction disorder | 12 (0.1) | 0.77 (0.17 - 3.44) | 0.77 (0.12) | 20 (0.07) | 1.33 (0.18 - 9.9) | 1.33 (0.08) |
| Wolff-Parkinson-White syndrome | 10 (0.09) | 0.26 (0.09 - 0.75) | 0.26 (7.17) | 3 (0.01) | Inf (NaN - Inf) | Inf (0.2) |
| Sinus arrest | 7 (0.06) | Inf (NaN - Inf) | Inf (0.9) | 39 (0.14) | 1.3 (0.31 - 5.37) | 1.3 (0.13) |
| Torsade de pointes | 6 (0.05) | Inf (NaN - Inf) | Inf (0.77) | 16 (0.06) | Inf (NaN - Inf) | Inf (1.06) |
| Long QT syndrome | 6 (0.05) | Inf (NaN - Inf) | Inf (0.77) | 9 (0.03) | 0.6 (0.08 - 4.72) | 0.6 (0.24) |
| Arrhythmia supraventricular | 5 (0.04) | Inf (NaN - Inf) | Inf (0.64) | 34 (0.12) | 2.26 (0.31 - 16.51) | 2.26 (0.68) |
| Early repolarisation syndrome | 5 (0.04) | 0.64 (0.07 - 5.49) | 0.64 (0.17) |  |  |  |
| Bifascicular block | 1 (0.01) | Inf (NaN - Inf) | Inf (0.13) | 16 (0.06) | Inf (NaN - Inf) | Inf (1.06) |
| Bundle branch block bilateral | 0 | - | - | 3 (0.01) | 0.2 (0.02 - 1.92) | 0.2 (2.41) |

**Supplementary Table 6.** Disproportionality analysis of arrhythmia-related AEs by sex group.

| MedDRA PT | Female (34536) | | |  | Male (23541) | | |
| --- | --- | --- | --- | --- | --- | --- | --- |
|  | N (%) | ROR (95%Cl) | PRR |  | N (%) | ROR (95%Cl) | PRR |
| Tachycardia | 16754 (48.51) | 2.57 (2.35-2.81) | 2.56 (449.35) |  | 6748 (28.66) | 2.44 (2.14-2.77) | 2.43 (198.16) |
| Atrial fibrillation | 4684 (13.56) | 2.31 (1.97-2.72) | 2.31 (108.19) |  | 5157 (21.91) | 2.37 (2.05-2.73) | 2.36 (145.23) |
| Cardiac flutter | 1935 (5.6) | 5.97 (3.99-8.93) | 5.97 (98.05) |  | 909 (3.86) | 6.12 (3.54-10.59) | 6.12 (54.94) |
| Extrasystoles | 1547 (4.48) | 3.69 (2.59-5.27) | 3.69 (59.7) |  | 857 (3.64) | 5.36 (3.16-9.09) | 5.36 (48.86) |
| Bradycardia | 1487 (4.31) | 0.92 (0.76-1.1) | 0.92 (0.83) |  | 1440 (6.12) | 1.2 (0.98-1.46) | 1.2 (3.29) |
| Cardiac arrest | 1436 (4.16) | 1.44 (1.14-1.81) | 1.44 (9.32) |  | 2170 (9.22) | 1.39 (1.17-1.65) | 1.39 (13.91) |
| Ventricular extrasystoles | 1233 (3.57) | 2.68 (1.91-3.77) | 2.68 (34.96) |  | 804 (3.42) | 2.71 (1.83-4) | 2.71 (27.13) |
| Sinus tachycardia | 1068 (3.09) | 1.65 (1.23-2.2) | 1.65 (11.67) |  | 549 (2.33) | 1.72 (1.17-2.51) | 1.72 (7.97) |
| Supraventricular tachycardia | 754 (2.18) | 1.8 (1.26-2.58) | 1.8 (10.59) |  | 390 (1.66) | 3.1 (1.71-5.65) | 3.1 (15.26) |
| Cardio-respiratory arrest | 621 (1.8) | 1.02 (0.75-1.38) | 1.02 (0.02) |  | 850 (3.61) | 1.43 (1.08-1.89) | 1.43 (6.37) |
| Supraventricular extrasystoles | 376 (1.09) | 2.32 (1.31-4.12) | 2.32 (8.72) |  | 283 (1.2) | 3.1 (1.53-6.25) | 3.1 (11.05) |
| Atrial flutter | 332 (0.96) | 2.46 (1.31-4.61) | 2.46 (8.39) |  | 533 (2.26) | 2.59 (1.62-4.15) | 2.59 (17.04) |
| Ventricular tachycardia | 297 (0.86) | 2.75 (1.36-5.55) | 2.75 (8.66) |  | 491 (2.09) | 3.31 (1.91-5.74) | 3.31 (20.38) |
| Bundle branch block left | 198 (0.57) | 3.66 (1.36-9.86) | 3.66 (7.59) |  | 169 (0.72) | 2.47 (1.09-5.57) | 2.47 (5.05) |
| Bundle branch block right | 182 (0.53) | 2.69 (1.11-6.55) | 2.69 (5.18) |  | 281 (1.19) | 2.05 (1.15-3.65) | 2.05 (6.19) |
| Cardiac fibrillation | 180 (0.52) | 2.22 (0.98-5.01) | 2.22 (3.89) |  | 155 (0.66) | 6.79 (1.68-27.38) | 6.79 (9.74) |
| Ventricular fibrillation | 178 (0.52) | 4.39 (1.4-13.75) | 4.39 (7.73) |  | 405 (1.72) | 3.94 (2.04-7.63) | 3.94 (19.32) |
| Atrioventricular block | 176 (0.51) | 2.61 (1.07-6.34) | 2.61 (4.81) |  | 198 (0.84) | 8.67 (2.15-34.9) | 8.67 (13.43) |
| Sinus bradycardia | 165 (0.48) | 0.76 (0.46-1.28) | 0.76 (1.07) |  | 225 (0.96) | 1.64 (0.92-2.93) | 1.64 (2.86) |
| Tachyarrhythmia | 135 (0.39) | 2.5 (0.92-6.75) | 2.5 (3.49) |  | 93 (0.4) | 4.07 (1-16.52) | 4.07 (4.54) |
| Atrioventricular block complete | 135 (0.39) | 5 (1.24-20.18) | 5 (6.3) |  | 176 (0.75) | 7.71 (1.91-31.05) | 7.7 (11.54) |
| Sinus arrhythmia | 114 (0.33) | 1.21 (0.56-2.59) | 1.21 (0.23) |  | 136 (0.58) | 3.97 (1.26-12.46) | 3.97 (6.52) |
| Atrioventricular block second degree | 99 (0.29) | 3.66 (0.9-14.85) | 3.66 (3.8) |  | 99 (0.42) | - | - |
| Atrial tachycardia | 97 (0.28) | 1.79 (0.66-4.88) | 1.79 (1.35) |  | 68 (0.29) | 1.49 (0.54-4.08) | 1.49 (0.61) |
| Tachycardia paroxysmal | 68 (0.2) | 5.03 (0.7-36.24) | 5.03 (3.18) |  | 24 (0.1) | 2.1 (0.28-15.53) | 2.1 (0.55) |
| Atrioventricular block first degree | 67 (0.19) | 2.48 (0.61-10.12) | 2.48 (1.71) |  | 106 (0.45) | 2.32 (0.85-6.3) | 2.32 (2.9) |
| Ventricular arrhythmia | 58 (0.17) | 4.29 (0.59-30.99) | 4.29 (2.48) |  | 64 (0.27) | - | - |
| Arrhythmia supraventricular | 57 (0.17) | 4.22 (0.58-30.47) | 4.22 (2.41) |  | 41 (0.17) | 3.59 (0.49-26.1) | 3.59 (1.82) |
| Sinus arrest | 25 (0.07) | 0.93 (0.22-3.91) | 0.93 (0.01) |  | 34 (0.14) | 1.49 (0.36-6.2) | 1.49 (0.3) |
| Conduction disorder | 21 (0.06) | 1.55 (0.21-11.55) | 1.55 (0.19) |  | 27 (0.11) | 0.59 (0.21-1.69) | 0.59 (0.99) |
| Torsade de pointes | 20 (0.06) | - | - |  | 10 (0.04) | - | - |
| Long QT syndrome | 14 (0.04) | - | - |  | 10 (0.04) | 0.88 (0.11-6.84) | 0.88 (0.02) |
| Wolff-Parkinson-White syndrome | 12 (0.03) | 0.44 (0.1-1.98) | 0.44 (1.19) |  | 15 (0.06) | 0.33 (0.11-0.99) | 0.33 (4.34) |
| Bifascicular block | 7 (0.02) | - | - |  | 14 (0.06) | 1.23 (0.16-9.32) | 1.23 (0.04) |
| Bundle branch block bilateral | 2 (0.01) | - | - |  | 4 (0.02) | 0.35 (0.04-3.13) | 0.35 (0.96) |
| Early repolarisation syndrome | 2 (0.01) | 0.15 (0.01-1.63) | 0.15 (3.27) |  | 6 (0.03) | - | - |

**Supplementary Table 7.** Disproportionality analysis of arrhythmia-related AEs by vaccine type.

| MedDRA PT | JANSSEN (2203) | | |  | MODERNA (15425) | | |  | PFIZER (41695) | | |
| --- | --- | --- | --- | --- | --- | --- | --- | --- | --- | --- | --- |
|  | N (%) | ROR (95%Cl) | PRR |  | N (%) | ROR (95%Cl) | PRR |  | N (%) | ROR (95%Cl) | PRR |
| Tachycardia | 854 (38.77) | 0.61  (0.57-0.66) | 0.61  (199.82) |  | 5866 (38.03) | 0.77  (0.75-0.79) | 0.77  (313.98) |  | 17335 (41.58) | 1.7  (1.66-1.75) | 1.7  (1500.77) |
| Atrial fibrillation | 356 (16.16) | 0.61  (0.55-0.68) | 0.61  (85.81) |  | 2964 (19.22) | 0.99  (0.95-1.03) | 0.99  (0.37) |  | 6747 (16.18) | 1.35  (1.29-1.4) | 1.35  (210.07) |
| Cardiac arrest | 212 (9.62) | 0.99  (0.86-1.13) | 0.99  (0.03) |  | 1020 (6.61) | 0.87  (0.81-0.94) | 0.87  (13.48) |  | 2446 (5.87) | 1.22  (1.14-1.3) | 1.22  (36.22) |
| Bradycardia | 161 (7.31) | 0.9  (0.77-1.05) | 0.9  (1.75) |  | 687 (4.45) | 0.66  (0.61-0.72) | 0.66  (91.46) |  | 2163 (5.19) | 1.46  (1.36-1.57) | 1.46  (103.32) |
| Cardio-respiratory arrest | 82 (3.72) | 0.93  (0.75-1.17) | 0.93  (0.36) |  | 380 (2.46) | 0.78  (0.69-0.87) | 0.78  (18.7) |  | 1029 (2.47) | 1.33  (1.2-1.48) | 1.33  (29.92) |
| Sinus tachycardia | 81 (3.68) | 0.86  (0.68-1.07) | 0.86  (1.87) |  | 390 (2.53) | 0.73  (0.65-0.82) | 0.73  (29.62) |  | 1160 (2.78) | 1.54  (1.39-1.7) | 1.54  (70.16) |
| Cardiac flutter | 78 (3.54) | 0.46  (0.37-0.58) | 0.46  (47.2) |  | 822 (5.33) | 0.95  (0.87-1.02) | 0.95  (1.88) |  | 2035 (4.88) | 1.58  (1.46-1.71) | 1.58  (135.96) |
| Ventricular extrasystoles | 53 (2.41) | 0.44  (0.33-0.58) | 0.44  (36.9) |  | 630 (4.08) | 1.04  (0.95-1.14) | 1.04  (0.74) |  | 1376 (3.3) | 1.33  (1.22-1.46) | 1.33  (40.53) |
| Extrasystoles | 52 (2.36) | 0.37  (0.28-0.48) | 0.37  (55.75) |  | 541 (3.51) | 0.69  (0.62-0.75) | 0.69  (60.96) |  | 1856 (4.45) | 2.13  (1.94-2.33) | 2.13  (281.56) |
| Supraventricular tachycardia | 47 (2.13) | 0.7  (0.52-0.93) | 0.7  (5.91) |  | 367 (2.38) | 1.08  (0.96-1.22) | 1.08  (1.62) |  | 748 (1.79) | 1.19  (1.06-1.34) | 1.19  (8.52) |
| Atrial flutter | 32 (1.45) | 0.63  (0.44-0.9) | 0.63  (6.71) |  | 265 (1.72) | 1.02  (0.89-1.18) | 1.02  (0.1) |  | 585 (1.4) | 1.33  (1.16-1.52) | 1.33  (16.6) |
| Sinus bradycardia | 26 (1.18) | 1.13  (0.76-1.68) | 1.13  (0.35) |  | 96 (0.62) | 0.73  (0.58-0.91) | 0.73  (7.64) |  | 276 (0.66) | 1.37  (1.12-1.67) | 1.37  (9.43) |
| Ventricular fibrillation | 25 (1.13) | 0.74  (0.5-1.11) | 0.74  (2.12) |  | 157 (1.02) | 0.87  (0.73-1.04) | 0.87  (2.24) |  | 408 (0.98) | 1.52  (1.28-1.81) | 1.52  (23.49) |
| Bundle branch block left | 22 (1) | 1.05  (0.68-1.62) | 1.05  (0.05) |  | 94 (0.61) | 0.81  (0.64-1.02) | 0.81  (3.23) |  | 255 (0.61) | 1.46  (1.18-1.81) | 1.46  (12.4) |
| Bundle branch block right | 20 (0.91) | 0.74  (0.47-1.16) | 0.74  (1.78) |  | 122 (0.79) | 0.83  (0.68-1.02) | 0.83  (3.22) |  | 327 (0.78) | 1.5  (1.24-1.81) | 1.5  (17.59) |
| Supraventricular extrasystoles | 19 (0.86) | 0.49  (0.31-0.77) | 0.49  (9.78) |  | 174 (1.13) | 0.84  (0.71-1) | 0.84  (3.87) |  | 474 (1.14) | 1.63  (1.39-1.92) | 1.63  (35.69) |
| Ventricular tachycardia | 15 (0.68) | 0.32  (0.19-0.54) | 0.32  (21.04) |  | 245 (1.59) | 1.06  (0.91-1.23) | 1.06  (0.59) |  | 536 (1.29) | 1.39  (1.2-1.61) | 1.39  (20.06) |
| Atrioventricular block first degree | 12 (0.54) | 1.23  (0.68-2.2) | 1.23  (0.47) |  | 51 (0.33) | 0.98  (0.71-1.35) | 0.98  (0.02) |  | 112 (0.27) | 1.19  (0.88-1.61) | 1.19  (1.3) |
| Atrioventricular block | 11 (0.5) | 0.5  (0.27-0.91) | 0.5  (5.4) |  | 121 (0.78) | 1.11  (0.9-1.38) | 1.11  (0.9) |  | 252 (0.6) | 1.32  (1.07-1.63) | 1.32  (6.95) |
| Sinus arrhythmia | 11 (0.5) | 0.75  (0.41-1.37) | 0.75  (0.88) |  | 43 (0.28) | 0.48  (0.35-0.67) | 0.48  (20.01) |  | 198 (0.47) | 2.2  (1.67-2.92) | 2.2  (32.17) |
| Atrioventricular block complete | 8 (0.36) | 0.44  (0.22-0.89) | 0.44  (5.46) |  | 80 (0.52) | 0.83  (0.64-1.07) | 0.83  (2.11) |  | 226 (0.54) | 1.78  (1.39-2.26) | 1.78  (22.22) |
| Atrioventricular block second degree | 7 (0.32) | 0.62  (0.29-1.32) | 0.62  (1.55) |  | 51 (0.33) | 0.84  (0.61-1.16) | 0.84  (1.09) |  | 142 (0.34) | 1.77  (1.3-2.4) | 1.77  (13.82) |
| Ventricular arrhythmia | 4 (0.18) | 0.56  (0.21-1.51) | 0.56  (1.35) |  | 20 (0.13) | 0.46  (0.29-0.74) | 0.46  (10.61) |  | 102 (0.24) | 2.95  (1.91-4.57) | 2.95  (25.91) |
| Sinus arrest | 3 (0.14) | 0.86  (0.27-2.74) | 0.86  (0.07) |  | 18 (0.12) | 0.99  (0.57-1.7) | 0.99  (0) |  | 38 (0.09) | 1.1  (0.66-1.82) | 1.1  (0.14) |
| Arrhythmia supraventricular | 2 (0.09) | 0.35  (0.09-1.41) | 0.35  (2.41) |  | 8 (0.05) | 0.21  (0.1-0.44) | 0.21  (21.54) |  | 90 (0.22) | 5.92  (3.16-11.07) | 5.92  (40.07) |
| Atrial tachycardia | 2 (0.09) | 0.2  (0.05-0.8) | 0.2  (6.46) |  | 43 (0.28) | 0.8  (0.57-1.12) | 0.8  (1.67) |  | 124 (0.3) | 1.73  (1.25-2.38) | 1.73  (11.16) |
| Cardiac fibrillation | 2 (0.09) | 0.1  (0.02-0.4) | 0.1  (16.09) |  | 66 (0.43) | 0.58  (0.45-0.76) | 0.58  (15.86) |  | 271 (0.65) | 2.65  (2.05-3.43) | 2.65  (59.68) |
| Tachyarrhythmia | 2 (0.09) | 0.15  (0.04-0.59) | 0.15  (9.84) |  | 50 (0.32) | 0.66  (0.49-0.91) | 0.66  (6.74) |  | 179 (0.43) | 2.27  (1.69-3.06) | 2.27  (30.78) |
| Bifascicular block | 2 (0.09) | 1.72  (0.4-7.34) | 1.72  (0.54) |  | 9 (0.06) | 1.71  (0.73-4) | 1.71  (1.56) |  | 11 (0.03) | 0.72  (0.31-1.67) | 0.72  (0.58) |
| Tachycardia paroxysmal | 1 (0.05) | 0.18  (0.03-1.3) | 0.18  (3.68) |  | 17 (0.11) | 0.53  (0.31-0.9) | 0.53  (5.8) |  | 76 (0.18) | 2.75  (1.68-4.5) | 2.75  (17.62) |
| Conduction disorder | 1 (0.05) | 0.31  (0.04-2.26) | 0.31  (1.49) |  | 8 (0.05) | 0.41  (0.19-0.87) | 0.41  (5.78) |  | 42 (0.1) | 2.17  (1.19-3.97) | 2.17  (6.63) |
| Torsade de pointes | - | - | - |  | 7 (0.05) | 0.72  (0.31-1.67) | 0.72  (0.59) |  | 23 (0.06) | 2.08  (0.93-4.65) | 2.08  (3.33) |
| Wolff-Parkinson-White syndrome | - | - | - |  | 5 (0.03) | 0.41  (0.16-1.06) | 0.41  (3.61) |  | 23 (0.06) | 1.39  (0.69-2.79) | 1.39  (0.85) |
| Long QT syndrome | - | - | - |  | 5 (0.03) | 0.62  (0.23-1.64) | 0.62  (0.95) |  | 19 (0.05) | 2.29  (0.91-5.74) | 2.29  (3.32) |
| Bundle branch block bilateral | - | - | - |  | 2 (0.01) | 0.99  (0.19-5.09) | 0.99  (0) |  | 4 (0.01) | 0.96  (0.22-4.31) | 0.96  (0) |
| Early repolarisation syndrome | - | - | - |  | 1 (0.01) | 0.31  (0.04-2.47) | 0.31  (1.38) |  | 7 (0.02) | 2.53  (0.53-12.19) | 2.53  (1.44) |

**Supplementary Table 8. Details of other vaccines included in the reference group for disproportionality analyses.**

| **Vaccine type** | **Description / representative vaccine names** | **N (%)** |
| --- | --- | --- |
| 6VAX-F | Six-vaccine combination product | 11 (0) |
| ADEN_4_7 | Adenovirus type 4 and type 7 vaccine | 83 (0.03) |
| ANTH | Anthrax vaccine | 383 (0.15) |
| BCG | Bacillus Calmette–Guérin vaccine | 24 (0.01) |
| CHIK | Chikungunya vaccine | 25 (0.01) |
| CHOL | Cholera vaccine | 67 (0.03) |
| COVID19 | COVID-19 vaccine records not included in the five target products, mainly unknown manufacturer | 8100 (3.19) |
| DF | Dengue vaccine | 63 (0.02) |
| DT | Diphtheria and tetanus vaccine | 71 (0.03) |
| DTAP | Diphtheria, tetanus, and acellular pertussis vaccine | 3905 (1.54) |
| DTAPH | DTaP and Hib combination vaccine | 1 (0) |
| DTAPHEPBIP | DTaP, hepatitis B, and inactivated poliovirus combination vaccine | 2221 (0.88) |
| DTAPIPV | DTaP and inactivated poliovirus vaccine | 4558 (1.8) |
| DTAPIPVHIB | DTaP, inactivated poliovirus, and Hib combination vaccine | 2576 (1.02) |
| DTOX | Diphtheria toxoid vaccine | 4 (0) |
| DTP | Diphtheria, tetanus, and pertussis vaccine | 41 (0.02) |
| DTPHEP | DTP and hepatitis vaccine | 1 (0) |
| DTPIHI | DTP, IPV, and Hib combination vaccine | 1 (0) |
| DTPPVHBHPB | DTP, polio, Hib, and hepatitis B combination vaccine | 1244 (0.49) |
| EBZR | Ebola Zaire vaccine | 56 (0.02) |
| FLU(H1N1) | Influenza A(H1N1) vaccine | 2 (0) |
| FLU3 | Trivalent seasonal influenza vaccines | 5526 (2.18) |
| FLU4 | Quadrivalent seasonal influenza vaccines | 25413 (10.02) |
| FLUA3 | Adjuvanted trivalent seasonal influenza vaccine | 504 (0.2) |
| FLUA4 | Adjuvanted quadrivalent seasonal influenza vaccine | 3869 (1.52) |
| FLUC3 | Cell-based trivalent seasonal influenza vaccine | 249 (0.1) |
| FLUC4 | Cell-based quadrivalent seasonal influenza vaccine | 4065 (1.6) |
| FLUN3 | Live attenuated trivalent influenza vaccine, nasal | 82 (0.03) |
| FLUN4 | Live attenuated quadrivalent influenza vaccine, nasal | 281 (0.11) |
| FLUR3 | Recombinant trivalent seasonal influenza vaccine | 104 (0.04) |
| FLUR4 | Recombinant quadrivalent seasonal influenza vaccine | 2313 (0.91) |
| FLUX | Seasonal influenza vaccine, no brand name or unspecified formulation | 7105 (2.8) |
| FLUX(H1N1) | Influenza A(H1N1) vaccine, unspecified formulation | 15 (0.01) |
| H5N1 | H5N1 influenza vaccine | 1 (0) |
| HBHEPB | Haemophilus influenzae type b and hepatitis B vaccine | 1 (0) |
| HEP | Hepatitis B vaccine | 7300 (2.88) |
| HEPA | Hepatitis A vaccine | 7641 (3.01) |
| HEPAB | Hepatitis A and hepatitis B combination vaccine | 1323 (0.52) |
| HEPATYP | Hepatitis A and typhoid combination vaccine | 1 (0) |
| HIBV | Haemophilus influenzae type b vaccine | 4793 (1.89) |
| HPV2 | Human papillomavirus bivalent vaccine | 5 (0) |
| HPV4 | Human papillomavirus quadrivalent vaccine | 845 (0.33) |
| HPV9 | Human papillomavirus 9-valent vaccine; e.g., Gardasil 9 | 8406 (3.31) |
| HPVX | Human papillomavirus vaccine, unspecified formulation | 73 (0.03) |
| IPV | Inactivated poliovirus vaccine | 2540 (1) |
| JEV1 | Japanese encephalitis vaccine | 132 (0.05) |
| JEVX | Japanese encephalitis vaccine, unspecified formulation | 5 (0) |
| LYME | Lyme disease vaccine | 1 (0) |
| MEA | Measles vaccine | 1 (0) |
| MEN | Meningococcal vaccine | 455 (0.18) |
| MENB | Meningococcal B vaccine | 4255 (1.68) |
| MENHIB | Meningococcal and Hib combination vaccine | 1 (0) |
| MMR | Measles, mumps, and rubella vaccine | 6841 (2.7) |
| MMRV | Measles, mumps, rubella, and varicella vaccine | 6803 (2.68) |
| MNP | Meningococcal polysaccharide vaccine | 17 (0.01) |
| MNQ | Meningococcal conjugate vaccine | 7070 (2.79) |
| MNQHIB | Meningococcal conjugate and Hib combination vaccine | 1 (0) |
| MU | Mumps vaccine | 2 (0) |
| OPV | Oral poliovirus vaccine | 2 (0) |
| PER | Pertussis vaccine | 1 (0) |
| PNC | Pneumococcal vaccine, unspecified formulation | 201 (0.08) |
| PNC10 | Pneumococcal conjugate vaccine, 10-valent | 3 (0) |
| PNC13 | Pneumococcal conjugate vaccine, 13-valent | 4788 (1.89) |
| PNC15 | Pneumococcal conjugate vaccine, 15-valent | 783 (0.31) |
| PNC20 | Pneumococcal conjugate vaccine, 20-valent | 4324 (1.7) |
| PNC21 | Pneumococcal conjugate vaccine, 21-valent | 89 (0.04) |
| PPV | Pneumococcal polysaccharide vaccine | 6861 (2.7) |
| RAB | Rabies vaccine | 917 (0.36) |
| RSV | Respiratory syncytial virus vaccine | 6518 (2.57) |
| RUB | Rubella vaccine | 1 (0) |
| RV1 | Rotavirus vaccine, monovalent | 1094 (0.43) |
| RV5 | Rotavirus vaccine, pentavalent | 3795 (1.5) |
| RVX | Rotavirus vaccine, unspecified formulation | 323 (0.13) |
| SMALL | Smallpox vaccine | 103 (0.04) |
| SMALLMNK | Smallpox and mpox vaccine | 2091 (0.82) |
| TBE | Tick-borne encephalitis vaccine | 11 (0) |
| TD | Tetanus and diphtheria vaccine | 1015 (0.4) |
| TDAP | Tetanus, diphtheria, and acellular pertussis vaccine | 9312 (3.67) |
| TDAPIPV | Tdap and inactivated poliovirus vaccine | 1 (0) |
| TTOX | Tetanus toxoid vaccine | 137 (0.05) |
| TYP | Typhoid vaccine | 1679 (0.66) |
| UNK | Vaccine not specified or unknown vaccine | 20039 (7.9) |
| VARCEL | Varicella vaccine | 8602 (3.39) |
| VARZOS | Zoster vaccine; e.g., Shingrix | 48999 (19.31) |
| YF | Yellow fever vaccine | 566 (0.22) |
